# Baseline Obesity Status Modifies Effectiveness of Adapted Diabetes Prevention Program Lifestyle Interventions for Weight Management in Primary Care

**DOI:** 10.1155/2013/191209

**Published:** 2013-12-04

**Authors:** Kristen M. J. Azar, Lan Xiao, Jun Ma

**Affiliations:** ^1^Palo Alto Medical Foundation Research Institute, 795 El Camino Real, Ames Building, Palo Alto, CA 94301, USA; ^2^Stanford Prevention Research Center, Department of Medicine, Stanford University School of Medicine, Stanford, CA, USA

## Abstract

*Objective*. To examine whether baseline obesity severity modifies the effects of two different, primary care-based, technology-enhanced lifestyle interventions among overweight or obese adults with prediabetes and/or metabolic syndrome. *Patients and Methods*. We compared mean differences in changes from baseline to 15 months in clinical measures of general and central obesity among participants randomized to usual care alone (*n* = 81) or usual care plus a coach-led group (*n* = 79) or self-directed individual (*n* = 81) intervention, stratified by baseline body mass index (BMI) category. *Results*. Participants with baseline BMI 35+ had greater reductions in mean BMI, body weight (as percentage change), and waist circumference in the coach-led group intervention, compared to usual care and the self-directed individual intervention (*P* < 0.05 for all). In contrast, the self-directed intervention was more effective than usual care only among participants with baseline BMIs between 25 ≤ 35. Mean weight loss exceeded 5% in the coach-led intervention regardless of baseline BMI category, but this was achieved only among self-directed intervention participants with baseline BMIs <35. *Conclusions*. Baseline BMI may influence behavioral weight-loss treatment effectiveness. Researchers and clinicians should take an individual's baseline BMI into account when developing or recommending lifestyle focused treatment strategy. This trial is registered with ClinicalTrials.gov NCT00842426.

## 1. Introduction

Obesity remains a pressing public health problem with adverse medical, psychological, social, and economical consequences. Nearly 70% of US adults are overweight (body mass index [BMI] in kg/m^2^  25 ≤ 30) or obese (BMI ≥30), with 36% obese [1]. More alarming still, the 6.3% prevalence of severely obesity (BMI ≥40) [1] is projected to increase by 130% over the next 2 decades [2]. Although bariatric surgery is the recommended treatment for severely obese individuals and/or moderately obese individuals (BMI 35 ≤ 40) with comorbidities [3], its implementation is limited by access, cost, recidivism, and complications [4, 5]. Only 1%-2% of obese people eligible for insurance coverage of surgical treatment receive it, compelling an urgent need of alternative treatment strategies for this subpopulation [6]. Weight loss medications have had limited effectiveness, some serious adverse effects, and limited uptake [7].

Emerging data find intensive lifestyle interventions—focusing on calorie-reduced, healthful eating, increased physical activity, and self-management skills training—can lead to clinically significant weight loss in the short [8–10] and long term [10, 11] among individuals with a BMI ≥35, who also achieve improvements in cardiovascular disease (CVD) risk factors even despite persistent, albeit reduced, obesity after intervention. Despite a recent and renewed interest in examining the efficacy of intensive behavior therapy for obesity within higher BMI subcategories [12], very few studies have evaluated the comparative effectiveness of evidence-based, empirical lifestyle interventions in real-world settings by baseline obesity status. Implementation of efficacious but resource-intensive, research-based lifestyle interventions into real-world settings remains a challenge. Efforts have been made to facilitate this process while retaining essential components of efficacious interventions [13]. However, these same efforts have resulted in wide variation in intervention setting, structure, intensity and form of contact, and resources required—and (unsurprisingly) they have produced mixed results regarding clinical effectiveness. Improved ability to implement targeted interventions for readily defined subgroups of the intended population may result in more efficient and effective use of resources.

The “Evaluation of Lifestyle Interventions to Treat Elevated Cardiometabolic Risk in Primary Care” (E-LITE) study was one of few pragmatic randomized controlled trials that successfully translated the Diabetes Prevention Program (DPP) lifestyle intervention into a primary care setting in the US. Published E-LITE data have demonstrated that two adapted, technology-enhanced DPP interventions (further described in Section 2)—one using a self-directed approach and the other a coach-led approach—were both superior to usual care, whereas the coach-led intervention was superior to the self-directed one, in promoting weight loss among overweight or obese adults with prediabetes and/or metabolic syndrome [15]. The primary aim of the current study was to examine whether changes in clinical measures of general and abdominal obesity differed by baseline BMI category when comparing the two interventions to usual care and to each other. We hypothesized that baseline BMI modified participant response to treatment such that participants with baseline BMI 35+ would benefit from the more structured, coach-led intervention, whereas those with lower starting BMI would respond to either coach-led or self-directed intervention.

## 2. Materials and Methods

Complete E-LITE trial protocol and methods were published previously [16]. Data collection occurred in 2009–2011. Below we describe methodological details relevant to this study.

### 2.1. Study Population and Measures

Participants were recruited (July 2009–June 2010) from a single primary care clinic that is part of a large multispecialty group practice in the San Francisco Bay Area. All data collection and intervention visits occurred at the clinic. Inclusion criteria included an age of at least 18 years, a BMI of at least 25, and the presence of prediabetes (defined by impaired fasting plasma glucose level of 100 to 125 mg/dL) or metabolic syndrome. Major exclusion criteria included serious medical or psychiatric conditions (e.g., stroke, psychotic disorder) or special life circumstances (e.g., pregnancy). Eligible and consenting overweight or obese adults with prediabetes and/or metabolic syndrome seen in primary care were randomized to receive usual care alone (*n* = 81) or usual care plus a coach-led (*n* = 79) or self-directed (*n* = 81) behavioral weight-loss intervention. Height was measured at baseline only, and weight and waist circumference were measured at baseline and at 3, 6, and 15 months. Measurements were taken in duplicate per standardized protocols [17, 18]. Body mass index was calculated. Change in BMI from baseline to 15 months was the trial primary outcome [15].

### 2.2. Intervention

The E-LITE study innovatively integrated the DPP-based Group Lifestyle Balance (GLB) core curriculum [13], which has been recognized by the Centers for Disease Prevention and Control's National Diabetes Prevention Program, with lifestyle coaching and self-management support via high-reach, affordable technologies. Both E-LITE interventions adopted the DPP's weight loss and physical activity goals [19] and delivered the GLB core curriculum for 12 weeks during the intensive treatment phase either through a self-directed, take-home DVD or coach-led, in-clinic small groups. The interventions also provided electronically mediated lifestyle coach contact and online self-monitoring of weight and physical activity goal attainment during a 12-month maintenance phase.  Table 1 summarizes the key components and features of the E-LITE self-directed and coach-led interventions.

### 2.3. Statistical Analyses

Baseline characteristics of each study group by baseline BMI category (25 ≤ 30, 30 ≤ 35, or 35+) were examined using analysis of variance (ANOVA) for continuous variables and Chi-square test for categorical variables. Between-group differences by baseline BMI category at 15 months were evaluated by intention-to-treat using all available data and tests of group by baseline BMI category interactions in repeated-measures mixed models. A separate model examined change in each of the 3 obesity outcome variables: BMI and percent body weight change for general obesity and waist circumference for abdominal obesity. The group by baseline BMI category interaction terms were significant for all three outcomes (*P* < 0.001). As in the main study [15], these models were adjusted for age, sex, race, and ethnicity, and missing data were handled directly through maximum likelihood estimation via mixed modeling. Model-based least-square mean changes and 95% confidence intervals (CIs) were obtained. All analyses were conducted using SAS, version 9.2 (SAS Institute Inc., Cary, North Carolina).

## 3. Results and Discussion

### 3.1. Results

**Figure 1:**
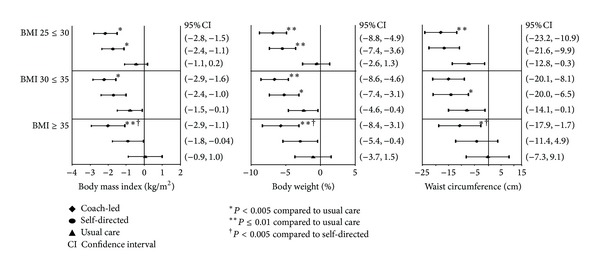
Outcomes by baseline body mass index category and intervention type.

Table 2 shows baseline sample characteristics, stratified by baseline BMI. No signifıcant differences in baseline characteristics were detected between participants in each treatment group. The coach-led intervention resulted in significantly greater mean reductions from baseline to 15 months in BMI (ranging from −2.0 kg/m^2^, 95% CI [−2.9 kg/m^2^, −1.1 kg/m^2^] in the BMI 35+ category to −2.2 kg/m^2^, 95% CI [−2.9 kg/m^2^, −1.6 kg/m^2^] in the BMI 30 ≤ 35 category) and percent body weight change (ranging from −6.8%, 95% CI [−8.8%, −4.9%] in the BMI 25 ≤ 30 category to −5.7%, 95% CI [−8.4%, −3.1%] in the BMI 35+ category) for all three baseline BMI categories, and in waist circumference for the BMI 25 ≤ 30 (−17.1 cm, 95% CI [−23.2 cm, −10.95 cm]) and 35+ (−9.78 cm, 95% CI [−17.9 cm, −1.7 cm]) categories (*P* < 0.05 versus usual care for all; Figure 1). The coach-led group achieved a mean percentage weight loss exceeding 5%, a commonly accepted threshold of clinically significant weight loss, and the upper 95% confidence limit was at least 3% weight loss, across the baseline BMI categories.

The self-directed intervention led to greater improvements in BMI (*P* = 0.03 versus usual care) only for the BMI 25 ≤ 30 category (−1.7 kg/m^2^, 95% CI [−2.4 kg/m^2^, −1.1 kg/m^2^]), in percentage weight loss for the BMI 25 ≤ 30 (−5.5%, 95% CI [−7.4%, −3.6%]; *P* < 0.0001 versus usual care) and 30 ≤ 35 (−5.2%, 95% CI [−7.4%, −3.1%]; *P* = 0.02 versus usual care) categories, and in waist circumference for the BMI 30 ≤ 35 category (−13.2 cm, 95% CI [−20.0 cm, −8.1 cm]; *P* = 0.03). In the self-directed group mean weight loss reached 5% only among those with a baseline BMI of <35. Moreover, reductions in BMI (*P* = 0.01), weight as percentage change (*P* = 0.04), and waist circumference (*P* = 0.04) were significantly greater in the coach-led versus self-directed intervention within the BMI 35+ category, whereas the two interventions did not differ significantly for any of the three obesity measures in the two lower BMI categories.

### 3.2. Discussion

Efficacy research has unequivocally shown that intensive, highly structured, individual lifestyle intervention lowers cardiometabolic risk [20]. The unabated obesity epidemic and its associated health problems and rising societal and economical burdens compel the urgency of adapting proven, albeit expensive, interventions into increasingly resource-limited real-world settings while striving to retain the effectiveness of the original treatment. The current findings show that the effects of the successful E-LITE coach-led and self-directed interventions in primary care differed by starting obesity status, suggesting that one size may not fit all when it comes to lifestyle interventions.

Notably participants with moderate or severe obesity (baseline BMI 35+) had greater reductions in all three obesity measures (BMI, percentage weight loss, and waist circumference) in the coach-led intervention, but not in the self-directed intervention, compared with usual care. They also responded more favorably to the coach-led intervention compared to the self-directed intervention. In contrast, overweight participants (baseline BMI 25 ≤ 30) had similar mean BMI and percent body weight reductions in the two active interventions, both of which were more effective than usual care. Similarly, the coach-led intervention had no apparent incremental benefit over the self-directed intervention for participants with a baseline BMI of 30 ≤ 35, although they led greater reductions in percentage weight loss and BMI (coach-led) or waist circumference (self-directed) compared with usual care. These results imply that the self-directed intervention can be an effective and efficient alternative to the coach-led intervention for overweight individuals with prediabetes and/or metabolic syndrome but that individuals with the same cardiometabolic risk factors who are moderately or severely obese may only benefit from the latter, more structured approach. These findings add to recent evidence that suggests a structured, intensive—and yet practical—lifestyle intervention is indicated for increased degree of obesity [10, 21], as opposed to a less structured, self-directed approach.

Previously, in the absence of empirical evidence, lifestyle intervention was thought to be ineffective in severely obese individuals [22] but has recently been recognized as a promising approach among this subpopulation [3, 8–12], especially given the risk of postoperative complications, recidivism, and limited reach of surgical options [4, 5, 8]. Modest weight loss for individuals who are overweight or obese (5%–10% reduction in total body weight) has been shown to produce health benefits such as improvement in blood pressure, cholesterol and dysglycemia [19, 23, 24] and was achieved among all participants in the coach-led intervention, including those whose baseline BMI was ≥35. A randomized controlled trial by Goodpaster et al. showed that an intensive behavioral weight-loss intervention was effective for severely obese adults and that modest weight reduction, even despite persistent severe obesity, significantly improved cardiovascular risk factors in this population [9]. A secondary analysis of data from the Look AHEAD trial found that nearly 40% of severely obese participants in the intensive, DPP-like lifestyle intervention lost ≥10% of initial weight at 1 year [8], and 26% were able to maintain this weight loss at year 4 [11].

This is the first study, to our knowledge, that suggests the potential effectiveness of a coach-led, technology-enhanced DPP translation intervention in reducing obesity among adults at high risk of Type 2 diabetes and CVD with a BMI of 35 or above. This is a growing segment of the overall population for which surgery is currently recommended; however, surgery cannot be the only solution to an epidemic. It is imperative that alternative strategies are developed that are effective, accessible, and affordable with potential for broad reach and impact. Equally important, our study suggests that the low cost, self-directed intervention can be a viable alternative to the coach-led intervention for high-risk adults with a BMI less than 35. The increased efficiency and reach of the self-directed intervention makes it an appealing public health intervention strategy.

Future studies are needed to explore factors that may modify or mediate the effectiveness of lifestyle interventions among persons with moderate or severe obesity. Potential effect modifiers include sociodemographic characteristics (e.g., sex, race/ethnicity, and education), comorbidity (e.g., severity of coexisting chronic conditions), and community/societal resources (e.g., accessibility of grocery stores or farmers market, neighborhood walkability, and social norms). Possible effect mediators include outcome expectancy, self-efficacy, social support, and self-monitoring, which are theory-based variables that have been shown to predict weight loss in diverse populations [25, 26].

The present findings should be interpreted with consideration of several study limitations. This was a secondary data analysis, and all findings warrant replication in future confirmatory research. The sample size for each BMI-by-treatment subgroup was small, and the trial duration was only 15 months. However, the effect size confidence intervals and data consistency across the three obesity outcome measures suggest that the E-LITE coach-led intervention may have clinically significant benefits beyond usual care for moderately and severely obese adults at high cardiometabolic risk that are worth further investigation in fully-powered, longer-term studies. Also, the generalizability of the current findings may be limited by a rather homogenous study sample in terms of race/ethnicity and socioeconomic status, and participants were recruited from a single primary care clinic. Future research is needed to confirm the generalizability of our findings to more ethnically and socioeconomically diverse populations.

## 4. Conclusion

In conclusion, baseline obesity status may influence behavioral weight loss treatment effectiveness. Less resource intensive approaches are perhaps adequate for individuals with lower baseline BMI in the overweight and obesity continuum, whereas the incremental benefit of more intensive, structured lifestyle change programs may not be evident except for those with higher BMI indicative of moderate or severe obesity. If confirmed in future definitive study, these findings would suggest that researchers and clinicians should take an individual's baseline BMI into account when developing or recommending a weight-loss treatment strategy. Understanding how to best allocate healthcare resources in weight-loss treatment may ultimately result in improved quality and affordability of obesity care.

## Figures and Tables

**Table 1:** E-LITE^a^ intervention: key components and features.

	Coach-led intervention	Self-directed intervention
(1) 12-week core curriculum sessions^b^	Clinic-based, small groups	Home-based DVD
(2) Online self-monitoring of weight and physical activity^c^	Preferably daily but at least twice weekly; coach routinely reviewed records	Preferably daily but at least twice weekly; coach did not routinely review records
(3) Personalized lifestyle coaching^d^	Proactive, coach-initiated	As needed, patient-initiated

^a^E-LITE: Evaluation of lifestyle interventions to treat elevated cardiometabolic risk in primary care.

^b^Diabetes Prevention Program (DPP) investigators at the University of Pittsburgh developed the Group Lifestyle Balance (GLB) program following the DPP trial [13]. Its curriculum is publicly available online [14].

^c^Via the American Heart Association's free, secure Heart360 Web portal (http://www.heart360.org/).

^d^Via secure provider-patient online messaging embedded in a fully functional electronic health record system. Coaches could view Heart360 patient self-monitoring records, which they reviewed regularly and used to tailor their ongoing progress feedback via secure messaging for participants in the coach-led intervention.

**Table 2:** Baseline characteristics of the study participants^a^.

Characteristic	All	Usual care	Coach-led	Self-directed	*F*/*χ* ^2^ (degree of freedom)	*P* value
Body mass index 25 ≤ 30						
Age, year	53.8 ± 10.5	53.7 ± 10.3	54.7 ± 10.9	53.1 ± 10.6	0.22 (2, 108)	0.80
Female, %	32.4	32.4	33.3	31.6	0.03 (2)	0.99
Race/ethnicity, %					3.29 (6)	0.77
Non-Hispanic white	72.1	75.7	66.7	73.7		
Asian/Pacific Islander	22.5	18.9	27.8	21.1		
Latino/Hispanic	4.5	5.4	5.6	2.6		
College level or above, %	97.2	97.2	94.3	100.0	2.22 (2)	0.33
Income, %					6.62 (6)	0.36
<$75,000	10.3	13.9	8.8	8.1		
$75,000–$124,999	30.8	27.8	41.2	24.3		
$125,000–$149,999	15.9	8.3	20.6	18.9		
$150,000+	43.0	50.0	29.4	48.6		
Weight, kg	83.8 ± 9.9	85.4 ± 9.1	82.6 ± 10.2	83.5 ± 10.6	0.77 (2, 108)	0.46
Waist, cm	98.8 ± 6.4	98.3 ± 6.4	98.0 ± 6.4	100.1 ± 6.4	1.14 (2, 108)	0.32
Fasting plasma glucose, mg/dL	100.3 ± 9.3	99.9 ± 9.5	101.4 ± 9.3	99.6 ± 9.2	0.39 (2, 108)	0.68
Prediabetes, %	56.8	62.2	61.1	47.4	2.08 (2)	0.35
Metabolic syndrome, %	80.2	67.6	88.9	84.2	5.81 (2)	0.06
Prediabetes and metabolic syndrome, %	36.9	29.7	50.0	31.6	3.93 (2)	0.14

Body mass index 30 ≤ 35						
Age, year	54.0 ± 10.8	54.5 ± 11.1	55.3 ± 12.9	52.3 ± 7.9	0.51 (2, 72)	0.60
Female, %	50.7	44.0	50.0	58.3	1.01 (2)	0.60
Race/ethnicity, %					5.25 (6)	0.51
Non-Hispanic white	80.0	80.0	80.8	79.2		
Asian/Pacific Islander	14.7	16.0	7.7	20.8		
Latino/Hispanic	4.0	4.0	7.7	0.0		
College level or above, %	97.3	96.0	96.2	100.0	0.97 (2)	0.62
Income, %					5.16 (6)	0.52
<$75,000	12.5	8.7	15.4	13.0		
$75,000–$124,999	23.6	30.4	23.1	17.4		
$125,000–$149,999	9.7	0.0	15.4	13.0		
$150,000+	54.2	60.9	46.2	56.5		
Weight, kg	94.2 ± 13.8	94.2 ± 12.9	97.2 ± 14.5	91.0 ± 13.8	1.26 (2, 72)	0.29
Waist, cm	106.7 ± 8.1	106.2 ± 8.9	109.2 ± 7.1	104.4 ± 7.9	2.35 (2, 72)	0.10
Fasting plasma glucose, mg/dL	100.3 ± 10.4	98.7 ± 8.6	100.4 ± 10.9	101.8 ± 11.6	0.52 (2, 72)	0.60
Prediabetes, %	50.7	44.0	50.0	58.3	1.01 (2)	0.60
Metabolic syndrome, %	88.0	92.0	80.8	91.7	1.97 (2)	0.37
Prediabetes and metabolic syndrome, %	38.7	36.0	30.8	50.0	2.06 (2)	0.36

Body mass index 35+						
Age, year	49.7 ± 10.1	47.4 ± 10.6	53.4 ± 8.5	48.6 ± 10.4	1.79 (2, 52)	0.18
Female, %	69.1	73.7	76.5	57.9	1.74 (2)	0.42
Race/ethnicity, %					2.58 (4)	0.63
Non-Hispanic white	87.3	78.9	94.1	89.5		
Asian/Pacific Islander	9.1	15.8	5.9	5.3		
Latino/Hispanic	3.6	5.3	0.0	5.3		
College level or above, %	98.2	94.7	100.0	100.0	1.93 (2)	0.38
Income, %					5.15 (6)	0.52
<$75,000	14.8	10.5	23.5	11.1		
$75,000–$124,999	22.2	26.3	29.4	11.1		
$125,000–$149,999	13.0	10.5	5.9	22.2		
$150,000+	50.0	52.6	41.2	55.6		
Weight, kg	113.4 ± 18.2	116.0 ± 20.0	111.6 ± 15.0	112.6 ± 19.4	0.29 (2, 52)	0.75
Waist, cm	120.1 ± 10.9	121.9 ± 11.7	117.3 ± 9.7	120.4 ± 11.4	0.78 (2, 52)	0.46
Fasting plasma glucose, mg/dL	98.7 ± 8.6	98.7 ± 8.7	98.7 ± 9.3	98.8 ± 8.3	0.00 (2, 52)	0.99
Prediabetes, %	54.5	52.6	58.8	52.6	0.18 (2)	0.91
Metabolic syndrome, %	98.2	100.0	94.1	100.0	2.28 (2)	0.32
Prediabetes and metabolic syndrome, %	52.7	52.6	52.9	52.6	0.0005 (2)	0.99

^a^Plus-minus values are means ± SD.
